# Formylation as a Chemical Tool to Modulate the Performance of Photosensitizers Based on Boron Dipyrromethene Dimers

**DOI:** 10.3390/ijms241411837

**Published:** 2023-07-23

**Authors:** Carolina Díaz-Norambuena, Edurne Avellanal-Zaballa, Alejandro Prieto-Castañeda, Jorge Bañuelos, Santiago de la Moya, Antonia R. Agarrabeitia, María J. Ortiz

**Affiliations:** 1 Departamento de Química Física, Facultad de Ciencia y Tecnología, Universidad del País Vasco-EHU, Apartado 644, 48080 Bilbao, Spain; carolina.diaz@ehu.eus (C.D.-N.); edurne.avellanal@ehu.eus (E.A.-Z.); alejandro.prietoc@ehu.eus (A.P.-C.); 2 Departamento Química Orgánica, Facultad de Ciencias Químicas, Universidad Complutense de Madrid, Ciudad Universitaria s/n, 28040 Madrid, Spain; santmoya@ucm.es (S.d.l.M.); agarrabe@quim.ucm.es (A.R.A.); 3 Sección Departamental de Química Orgánica, Facultad de Óptica y Optometría, Universidad Complutense de Madrid, Arcos de Jalón 118, 28037 Madrid, Spain

**Keywords:** BODIPY dimers, fluorescence, PDT, photosensitizers, singlet oxygen

## Abstract

Heavy-atom-free photosensitizers are envisioned as the next generation of photoactive molecules for photo-theragnosis. In this approach, and after suitable irradiation, a single molecular scaffold is able to visualize and kill tumour cells by fluorescence signalling and photodynamic therapy (PDT), respectively, with minimal side effects. In this regard, BODIPY-based orthogonal dimers have irrupted as suitable candidates for this aim. Herein, we analyse the photophysical properties of a set of formyl-functionalized BODIPY dimers to ascertain their suitability as fluorescent photosensitizers. The conducted computationally aided spectroscopic study determined that the fluorescence/singlet oxygen generation dual performance of these valuable BODIPY dimers not only depends on the BODIPY-BODIPY linkage and the steric hindrance around it, but also can be modulated by proper formyl functionalization at specific chromophoric positions. Thus, we propose regioselective formylation as an effective tool to modulate such a delicate photonic balance in BODIPY-based dimeric photosensitizers. The taming of the excited-state dynamics, in particular intramolecular charge transfer as the key underlying process mediating fluorescence deactivation vs. intersystem crossing increasing, could serve to increase fluorescence for brighter bioimaging, enhance the generation of singlet oxygen for killing activity, or balance both for photo-theragnosis.

## 1. Introduction

**Figure 1:**
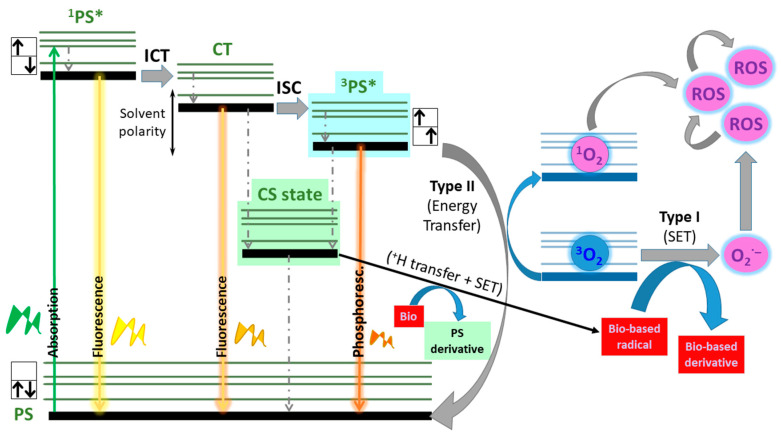
Cartoon of the main light-induced photophysical processes taking place in photo-theragnostic PSs involving SOC-ISC. Non-radiative relaxation processes are indicated with dotted lines.

Photodynamic therapy (PDT) has emerged as an alternative non-invasive treatment for cancer and other diseases [1,2,3,4,5,6,7,8]. This approach consists of the generation of cytotoxic reactive oxygen species (ROS), predominantly singlet oxygen (^1^O_2_), solely after irradiation of a proper photosensitizer (PS) of molecular oxygen with light of a specific wavelength. This photosensitizer should have a high enough intersystem crossing (ISC) probability to populate a triplet state (^3^PS*), the bottleneck channel for the ulterior generation of ^1^O_2_ via the Type I mechanism for ROS generation (Figure 1) [9,10,11]. Alternatively, ROS photo-generation can occur via the Type II mechanism, where a more complex process involving single-electron transfer (SET) from a sufficiently charge-separated (CS) state to oxygen, together with a proton transfer to a close biological specie, is involved (Figure 1). The key ISC can be easily promoted by decorating the PS with heavy atoms [12]. However, this functionalization induces high dark toxicity probability, short triplet lifetimes, and reduced photostability [13]. To offset such shortcomings, different alternatives are being currently explored to mediate ISC without heavy-atom participation. The main strategies are promoting photo-induced charge transfer processes to allow spin–orbit charge transfer ISC (SOC-ISC) [14,15,16], minimizing the singlet–triplet energy gap [17], increasing the n-π* character of the electronic transition (e.g., via thionation) [18], using π-conjugated twisted chromophoric systems enabling symmetry-breaking charge transfer (SBCT) and ISC [19,20], or radical-enhanced ISC [21]. As a result, smart heavy-atom-free PSs are currently being designed and reported [22,23,24,25,26]. A proper modulation of ISC probability is also valuable to retain a certain fluorescence capability along with triplet manifold population (Figure 1). This photo-induced dual behaviour can serve to generate ROS for cancer PDT and to simultaneously assess such a therapy by fluorescence bioimaging [27,28]. Indeed, small all-organic monochromophoric PSs endowed with such challenging photo-theragnostic ability embody the next generation of advanced agents for fighting aggressive cancers.

**Figure 2:**
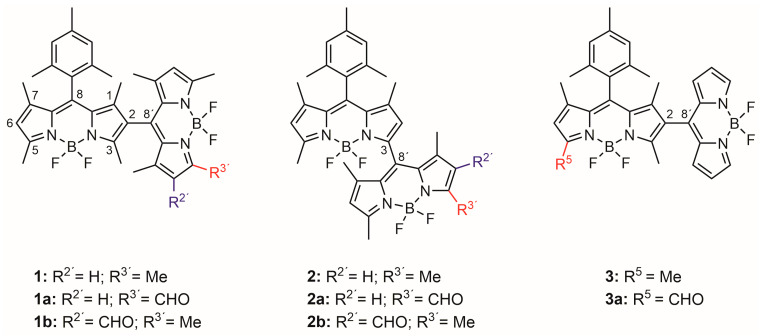
Studied non-formylated (**1**–**3**) and formylated (**1a,b**, **2a,b**, and **3a**) BODIPY dimers as potential fluorescent PSs for photo-theragnostic purposes.

The renowned BOron DIPYrromethene (BODIPY) chromophore has arisen as a suitable molecular scaffold to design heavy-atom-free PSs for photo-theragnosis [29,30,31]. At first sight, it is not suitable as a molecular PS, since its chromophoric core stands out for its high fluorescence response and almost null ISC probability [32]. However, its synthetic accessibility and functionalization, readily available through workable reaction routes, enable a wide structural diversity [33], which at the same time serves to tailor the photophysical properties of BODIPY dye [34]. In other words, it is feasible to induce new photophysical pathways to enhance the ISC viability, just by adjusting the molecular design in terms of choosing a proper substitution pattern [35]. In this regard, directly linked BODIPY dimers have been reported as efficient PSs of molecular oxygen (Figure 2). These heavy-atom-free PSs are actually in the spotlight owing to their inherent advantages related to the lack of heavy atoms. Indeed, the incorporation of heavy atoms enhances the dark toxicity, worsens the PS photostability, and shortens the triplet-state lifetimes [13]. In fact, heavy-atom-free BODIPY dimers are envisaged as the next generation of smart PSs to address such drawbacks and fit the clinical requirements for conducting PDT [36,37]. Such dimers usually involve *meso* (8) BODIPY positions in the BODIPY-BODIPY linkage (mainly 2-8′ and 3-8′ linkages), owing to the known sensitivity of BODIPY’s photophysics to structural modifications at this position, although other geometries have also been tested [13,22,23,24,38,39]. In these BODIPY dimers, the usually induced orthogonal disposition of the BODIPY subunits is able to induce SBCT, facilitating ISC [40]. The elucidation of this mechanism allowing triplet-state population has been the subject of controversy, with the SOC-ISC being the most commonly accepted pathway [14,15,16], although other mechanisms cannot be fully excluded [41,42]. For instance, single fission has been shown to be involved in J-type BODIPY dimers in the solid state [43].

Recently, we have tested and evaluated the suitability of a number of synthetically accessible and heavy-atom-free BODIPY dimers and trimers as PDT PSs (Figure 2) [44]. We found that, according to the precedents [45], the 2-8′ dimer **1** was able to exhibit efficient ^1^O_2_ photo-generation (^1^O_2_ photo-generation quantum yield, *ϕ^Δ^*, higher than 40% regardless of the polarity of the media). In spite of the promoted ISC, this dimer was able to retain a reasonable fluorescence emission. Obviously, its fluorescence quantum yield (*ϕ*_fl_) decreased in polar media, owing to the ongoing ICT, but values as high as 45% were registered. Therefore, the dual photonic performance of **1** supported its suitability as a photo-theragnostic PS. Interestingly, the 3-8′ linkage of the related BODIPY dimer **2** induced a higher ICT probability. As a result, the ISC probability was consequently enhanced, and a high *ϕ^Δ^* was recorded (up to 96%). However, the fluorescence response was severely quenched by the efficient ongoing ICT, making **2** only suitable as a PS for PDT, but not for photo-theragnosis.

Herein, we propose a simple and effective strategy to modulate the dual performance (fluorescence vs. singlet oxygen generation) in these kinds of 2-8′ and 3-8′ BODIPY dimers via regioselective formylation at a single chromophoric subunit. Such formylated dimers were reported previously by us as key precursors of BODIPY trimers [44]. However, we have recently realized that such a regioselective modification can be very appealing to trigger the performance of BODIPY dimers as photo-theragnostic PSs. Hereafter, we analyse in depth the impact of such BODIPY derivatization at different chromophoric positions (α and β pyrrolic positions) in the fluorescence response and ^1^O_2_ generation capability of BODIPY dimers involving 2-8′ or 3-8′ BODIPY-BODIPY linkages (see dimers **1** and **3**, and **2**, respectively, in Figure 2) and differential steric overcrowding around the junction (see dimers **1** vs. **3** in Figure 2). The purpose of this study is to evaluate the suitability of constructing formylated BODIPY dimers as a simple chemical tool to enhance their singlet oxygen photo-generation for PDT applications, or to ameliorate their fluorescence response for photo-theragnosis, regardless of the geometrical arrangement of the starting dimer.

## 2. Results and Discussion

The synthetic details and the corresponding structural characterization of all the studied dimers (Figure 2) were previously reported by us [44]. The linking typology (2-8′ vs. 3-8′) of the dimer has a marked impact on the photophysical signatures. Thus, the 2-8′ dimer **1** featured a strong absorption at 510 nm (Figure S1 in Supplementary Materials), resulting from the sum of the individual, local excitation (LE) of each BODIPY subunit (see HOMO-1→LUMO and HOMO→LUMO+1 transitions in Figure S2 in Supplementary Materials), as well as a solvent-dependent emission at 525 nm (Table 1 and Table S1 in Supplementary Materials). The ongoing SBCT progressively quenched the emission in polar media (*ϕ*_fl_ from 46% to just 3%, Table S1 in Supplementary Materials), but in turn it promoted triplet-state population (SOC-ISC), rendering a high ^1^O_2_ generation (*ϕ*^Δ^ between 40 and 95% depending on the media, Table S1 in Supplementary Materials). On the other hand, in the 3-8′ dimer **2**, the impact of the ICT was more marked. Thus, the molar absorption decreased (Figure S3 in Supplementary Materials) and the fluorescence response was almost negligible even in low-polarity solvents (Table 1 and Table S1 in Supplementary Materials). This kind of BODIPY-BODIPY arrangement provided more ICT character to the absorption transition (i.e., a more electronically forbidden character when compared to the allowed LE one), as suggested theoretically by the increased contribution of the HOMO→LUMO transition to the main absorption (Figure S4 in Supplementary Materials) [44]. Moreover, a weak long-wavelength emission at 596 nm, assigned to its own ICT emission, was detected following the quenched LE-emission band at 529 nm (Figure S3 in Supplementary Materials). Such an enhancement and stabilization of the charge separation by the solvent polarity completely removed the fluorescence emission from both excited states, ICT and LE, but still allowed a high ^1^O_2_ generation, except in the highly polar media (Table S1 in Supplementary Materials). It is likely that, in acetonitrile, the charge separation was so stabilized by the solvent polarity that the charge recombination required to reach the triplet manifold was hampered, yielding no measurable ^1^O_2_ emission. Nonetheless, we should bear in mind that determining *ϕ*^Δ^, by measuring ^1^O_2_ phosphorescence, is more difficult in acetonitrile, due to the short lifetime of ^1^O_2_ in such media. Therefore, whereas dimer **1** can be considered a fluorescent PS, dimer **2** cannot.

**Figure 3:**
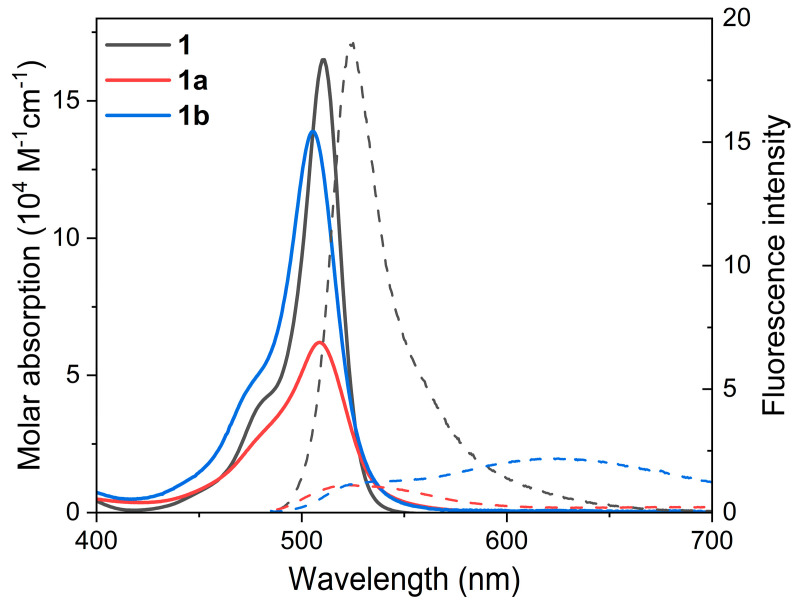
Absorption and fluorescence (dashed and scaled by fluorescence efficiency) spectra of the studied 2-8′ BODIPY dimers (**1** and its monoformylated derivatives **1a** and **1b**) from diluted solutions in chloroform. The corresponding spectra in other solvents of different polarity are collected in Figure S1, in Supplementary Materials.

**Figure 4:**
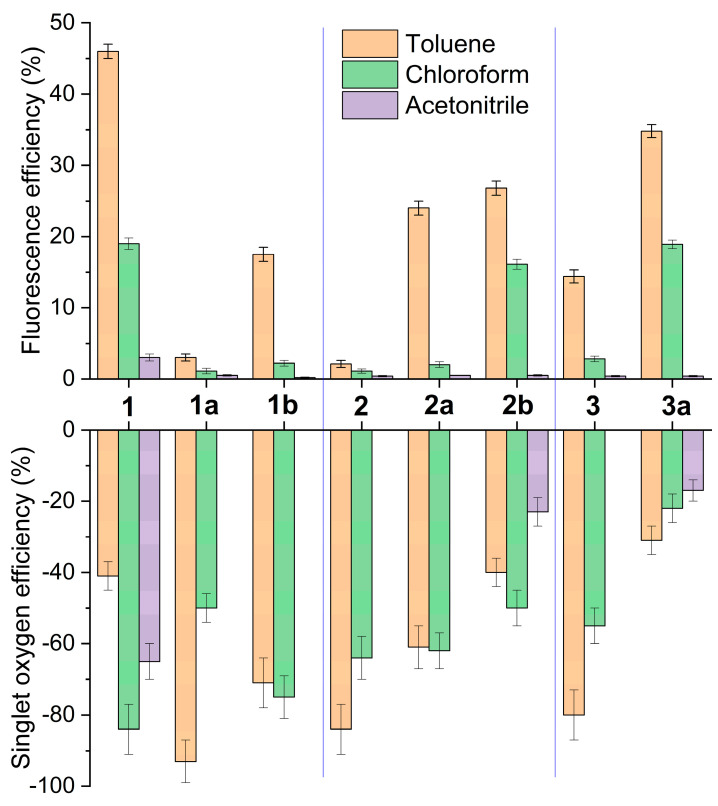
Evolution of the fluorescence and ^1^O_2_ photo-generation efficiencies of all the studied BODIPY dimers in representative solvents of different polarities.

With the aim of investigating the possible modulation of the photophysical signatures of BODIPY dimers **1**–**2**, we considered their regioselective monoformylation to be a feasible tool (Figure 2). BODIPY dimer monoformylation implies that the involved chromophoric subunits are no longer similar, owing to the moderate electron-withdrawing electronic effect exerted by the formyl group when compared to hydrogen or methyl. We hypothesized that this loss of similarity could alter the ICT probability and the ensuing ISC, thereby modulating the balance between fluorescence and ^1^O_2_ photo-generation. Formylation at position 2′ of the 2-8′ dimer **1**, to generate **1b**, quenched the fluorescence response (Table 1). Such a trend was even more marked upon formylation at position 3′ (**1a**), where not only the fluorescence efficiency was further reduced, but also the absorption probability was clearly lowered (Figure 3). In other words, BODIPY dimer monoformylation implies an increase in the CT character of the absorption, as predicted theoretically in terms of a higher contribution of the HOMO→LUMO transition for these dimers and a higher stabilization of the corresponding CT states upon excitation, which leads to an efficient solvent-driven quenching of the LE emission (Figure 4). Indeed, a broad and weak red-shifted emission from the ICT was recorded at 625 nm in chloroform for **1b**, and at 605 nm in toluene for **1a** (Figure S1 in Supplementary Materials). The decay curves of these formylated dimers, monitored at the ICT maximum, showed a higher contribution of the fast lifetimes than at the LE maximum in those media where dual emission was detected (Table 1). Thus, there is an optimal polarity to detect the ICT emission depending on the dimer’s molecular structure. Beyond such a polarity point, the ICT fluorescence is completely lost, and just a dim LE emission is detected (Figure 3 and Figure S1 in Supplementary Materials), likely because the CT evolves into a “dark” charge-separated CS state. In agreement, with a higher ICT population upon formylation, ^1^O_2_ photo-generation is very efficient in apolar media for these formylated dimers (up to 93% for **1a**), but it drops almost to null in polar media, owing to the non-radiative dissipation provided by the stabilized charge separation (Figure 4). Once again, as occurred with fluorescence, the solvent polarity triggers the generation of ^1^O_2_, and there is an optimal solvent polarity optimizing it, which depends on the dimer structure: chloroform for **1b** (75%) and toluene for **1a** (93%), the latter being the one with the higher ICT character. Further increasing the solvent polarity beyond such optimal points implies that the non-radiative decay of the CS prevails. Therefore, in dimer **1**, as a representative example of the 2-8′ BODIPY dimers, regioselective monoformylation worsens its performance as a fluorescent PS for ^1^O_2_ generation.

**Figure 5:**
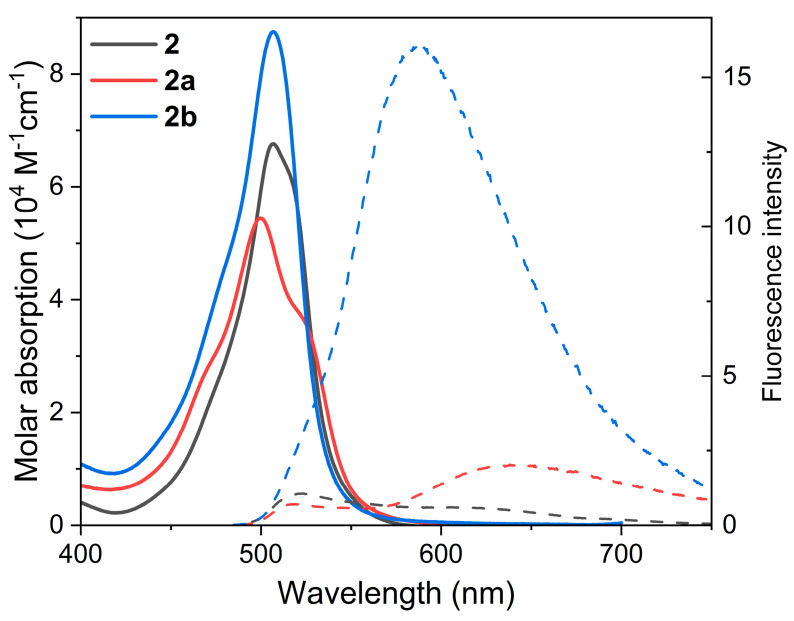
Absorption and fluorescence (dashed and scaled by fluorescence efficiency) spectra of the 3-8′ BODIPY dimer **2** and its monoformylated derivatives (**2a** and **2b**) from diluted solutions in chloroform. The corresponding spectra in other solvents of different polarities are collected in Figure S3, in Supplementary Materials.

The same regioselective monoformylations were conducted on the 3-8′ BODIPY dimer **2** (Figure 2) and different photophysical trends were noted (Table 1). In all cases, the formylation ameliorated the fluorescence response (Figure 4). Thus, formylation at position 3′ (**2a**) clearly increased the fluorescence efficiency of **2** in apolar media, but such enhancement was even more prominent and extended to polar media upon formylation at position 2′ (**2b**), suggesting a softened ICT quenching of the emission. Once again, the key role of solvent polarity in ICT stabilization depending on the molecular geometry is unambiguously stated. Thus, in this set of dimers, the absorption showed a long-wavelength shoulder, and the fluorescence profile was dominated by the ICT emission (Figure 5 and Figure S3 in Supplementary Materials). Formylation at position 2´ (**2b**) allows the ICT contribution in polar chloroform to be optimized, since not only the molar absorption increased in this solvent (Figure 5), but also the fluorescence was ameliorated (Figure 4). Indeed, the corresponding computed frontier molecular orbitals, albeit located mainly in one of the BODIPY subunits, are spread over the whole dimer (Figure S4 in Supplementary Materials). Such a trend is clearly visualized in HOMO and HOMO-1 of **2a**, as well as in LUMO and LUMO-1 of **2b**. Note that the emission of these formylated dimers is well separated from the absorption, suggesting that it holds an ICT character rather than LE (Figure 5 vs. Figure 3). Such ameliorated fluorescence response (*ϕ*_fl_ up to 27%) brings a lower ^1^O_2_ photo-generation, but the latter is high enough to make the formylated dimers **2a** and **2b** candidates good PSs for ^1^O_2_ generation (Figure 4). Indeed, dimer **2b** is able to exhibit a reasonable *ϕ*^Δ^ regardless of the solvent polarity (23–50%), sustaining that the key point in modulating BODIPY dimers for photo-theragnostic performance is to balance their ICT probabilities, as the bottleneck allows the triplet state to be reached and/or render the fluorescence signal. Therefore, regioselective monoformylation at position 3 is a simple but effective chemical strategy to transform a 3-8′ BODIPY-dimer PDT PS into a fluorescent PS for photo-theragnosis.

**Figure 6:**
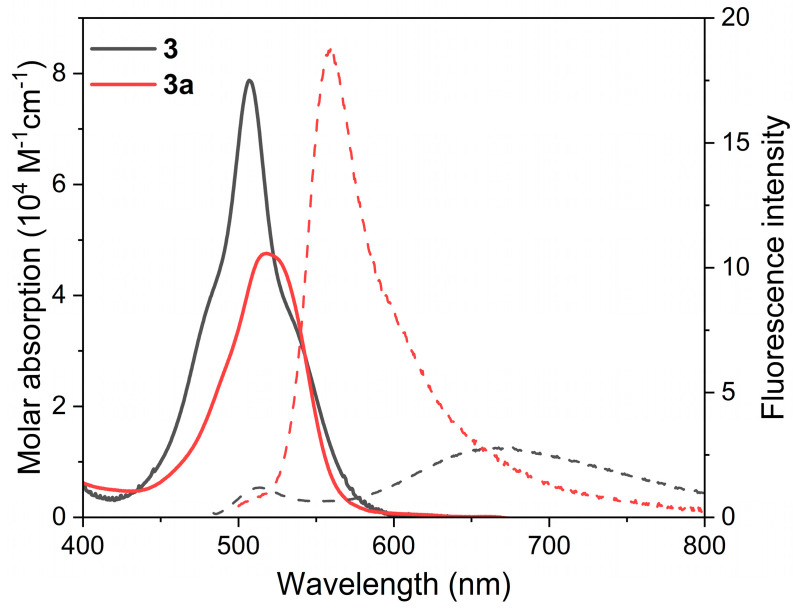
Absorption and fluorescence (dashed and scaled by their fluorescence efficiency) spectra of conformationally flexible 2-8′ BODIPY dimers (**3** and its formylated derivative **3a**) from diluted solutions in chloroform. The corresponding spectra in other solvents of different polarities are collected in Figure S5, in Supplementary Materials.

Another way to modulate the ICT state is by acting on the twisted relative disposition (dihedral angle) of the involved BODIPY chromophores [46,47,48]. Hitherto, BODIPY positions close to the BODIPY-BODIPY linkage were methylated, therefore inducing an orthogonal disposition of the BODIPY chromophores that ensures ICT-mediated ISC via enhanced SBCT. To investigate the influence of such a dihedral angle, and hence the ICT induction, the methyl groups were removed in just one of the chromophoric subunits of 2-8′ BODIPY dimer **1** to generate dimer **3** (see Figure 2). Compared to dimer **1**, in **3** both the absorption and emission probabilities were clearly reduced, suggesting a higher ICT probability (Table 1). In fact, the absorption profile of **3** was broader (Figure 6 and Figure S5 in Supplementary Materials). This fact can be attributed to the higher conformational freedom of **3**, which enables some degree of electronic coupling between the BODIPY subunits, as suggested by the computed frontier molecular orbitals, which show a more extended electron density pattern than in the case of related **1** (cf. Figures S6 and S2 in Supplementary Materials). In fact, the higher ICT population in **3** allows one to record, even in apolar media, the red-shifted weak emission from its ICT (Figure 6). Further increasing the solvent polarity quenches the whole emission of **3**, finally rendering a residual LE emission (Figure 4 and Figure S5 in Supplementary Materials). Accordingly, the higher *ϕ*^Δ^ was detected in toluene for **3** (80%), whereas it was in chloroform for **1** (84%), and ^1^O_2_ photo-generation was silent in polar media for **3** (Figure 4). Therefore, the reduction in the steric hindrance by an alkyl-alkyl (methyl-methyl) clash around the BODIPY-BODIPY linkage reduces the fluorescent capability of the 3-8′ BODIPY-dimer PS.

To investigate the possible further tuning of the photophysical properties of highly flexible dimer **3**, it was regioselectively monoformylated at position 5 to generate **3a**. It must be taken into account that related, but constrained, the monoformylated 2-8′ dimer **1a** was poorly fluorescent. However, in less-crowded **3a**, the formylation was profitable from a fluorescent point of view (Table 1). Thus, this BODIPY dimer still undergoes ICT, but its fluorescence-quenching effect is less strong than in the case of related **1a**, enabling the recording of quite bright fluorescence signals (Figure 6) in different solvents (*ϕ*_fl_ up to 35%, Figure 4). Indeed, the computed key frontier molecular orbitals of **3a** span along the whole dimer (Figure S6 in Supplementary Materials). In contrast, the singlet oxygen generation decreased but it was still high enough to be applied in therapy (*ϕ*^Δ^ around 20–30%, see Figure 4). Therefore, in this case (flexible 2-8′ BODIPY dimer), formylation allows fluorescent PSs to be obtained for photo-theragnosis.

**Figure 7:**
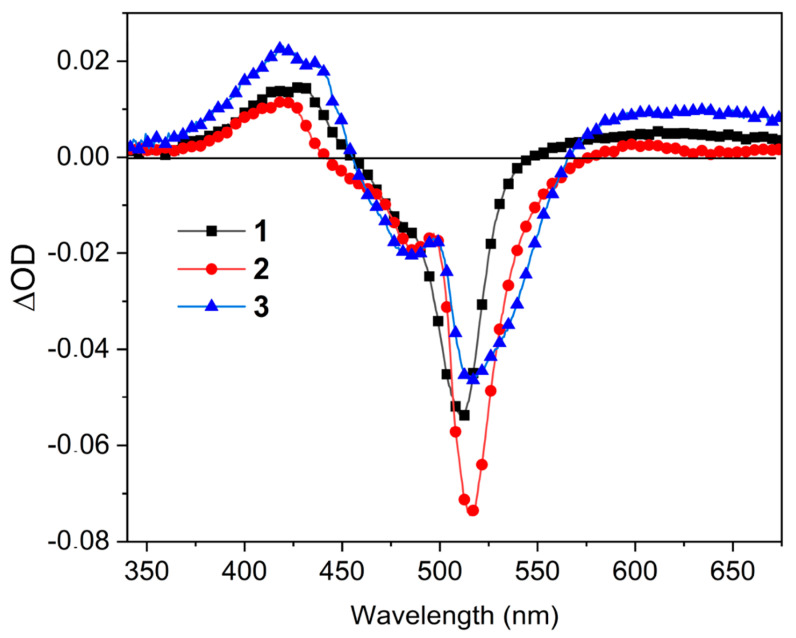
Transient absorption spectra of representative BODIPY dimers (**1**–**3**) in chloroform under nitrogen-saturated atmosphere. The corresponding spectra for the corresponding formylated dimers are collected in Figure S7, in Supplementary Materials.

**Figure 8:**
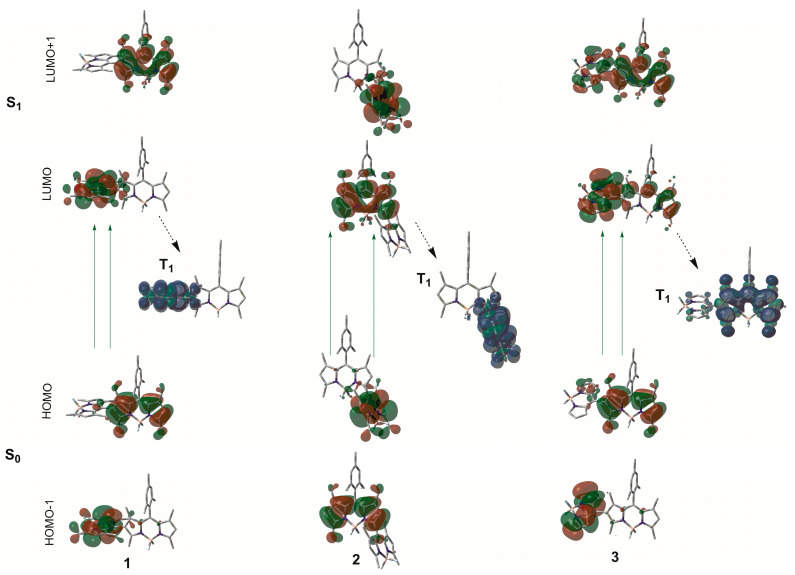
CAM-B3LYP/6-311G* computed electron density contour maps (at the corresponding singlet ground-state S_0_-optimized geometry) and spin density isosurfaces (at the corresponding triplet-state T_1_-optimized geometry) of representative BODIPY dimers (**1**–**3**). The spin density of T_1_ state for the corresponding formylated dimers are collected in Figure S8 in Supplementary Materials.

To identify the T_1_ state populated by all the studied BODIPY dimers we used laser flash photolysis. The recorded nanosecond-resolved transient absorption spectra (ns-TAS) are plotted in Figure 7 and Figure S7 in the Supplementary Materials. All the spectra showed a negative bleaching band, which matches the maximum of the ground-state absorption. This band is flanked by a positive short-wavelength one peaked at 425 nm, as well as by a flat and broad long-wavelength positive band corresponding to the T_1_-T_n_ absorptions. The decay of the positive signal was fitted to a monoexponential function, suggesting that only one transient state (T_1_) is populated, with triplet lifetimes, *τ*^T^, ranging from 110 μs to 220 μs (Table 1), which are long enough for efficient triplet oxygen to singlet oxygen photosensitizing. Indeed, a general rule can be established from the recorded BODIPY dimer behaviours; the higher the *ϕ*^Δ^ (84% and 75% in **1** and **1b**, respectively), the longer the *τ*^T^ (220 μs and 215 μs, respectively). In air-saturated solutions, such lifetimes decreased to hundreds of ns, whereas the profile and position of the ns-TAS spectral bands remained similar. This oxygen-induced quenching supports that the positive bands of the ns-TAS are due to T_1_-T_n_ absorptions. The T_1_ state was also theoretically studied by means of their corresponding spin-density isosurfaces (see Figure 8 and Figure S8 in Supplementary Materials). In most of them, the computed T_1_ spin density was located in one BODIPY subunit, in particular at the non-mesithylated BODIPY subunit; that is, at a BODIPY moiety connected through its 8 position, regardless of the BODIPY-BODIPY linkage (2-8′ or 3-8′) (e.g., see Figure 8). The only exception to this trend is that exhibited by the formylated 2-8′ dimer **1b**, where the spin density is shared by both BODIPYs (Figure S8 in Supplementary Materials).

## 3. Materials and Methods

### 3.1. Spectroscopic Techniques

The photophysical properties were registered from diluted solutions (around 2 × 10^−6^ M), prepared by adding the corresponding solvent to the residue from the adequate amount of a concentrated stock solution in acetone, after vacuum evaporation of this solvent. UV-Vis absorption and fluorescence spectra were recorded on a Varian model CARY 4E spectrophotometer and an Edinburgh Instruments spectrofluorometer (model FLSP 920), respectively. Fluorescence quantum yields (*ϕ*_fl_) were obtained using PM567 (*ϕ_fl_* = 0.84 in ethanol) in the spectral window 500–600 nm, cresyl violet (*ϕ*_fl_ = 0.54 in methanol) in the spectral window 600–650 nm, and zinc phthalocyanine (*ϕ*_fl_ = 0.30 in toluene with 1% of pyridine) in the 660–750 nm window, as references, from corrected spectra (detector sensibility to the wavelength). The values were corrected by the refractive index of the solvent. Radiative decay curves were registered with the time-correlated single-photon counting technique as implemented in the aforementioned spectrofluorometer. Fluorescence emission was monitored at the maximum emission wavelengths after excitation by means of a Fianium pulsed laser (time resolution of picoseconds) with tunable wavelength. The fluorescence lifetime (*τ_fl_*) was obtained after the deconvolution of the instrumental response signal from the recorded decay curves by means of an iterative method. The goodness of the exponential fit was controlled by statistical parameters (chi-square, Durbin–Watson, and the analysis of the residuals).

Nanosecond transient absorption spectra (ns-TAS) were recorded on an LP 980 laser flash photolysis spectrometer (Edinburgh Instruments, Livingston, UK). Samples were excited by a nanosecond pulsed laser (Nd:YAG laser, LOTIS TII 2134) operating at 1 Hz and a pulse width of ≥7 ns, coupled to an OPO which allows the selection of the excitation wavelength. The transient signals were recorded on single detector (PMT R928P), oscilloscope for kinetic traces, and ICCD detector DH320T TE cooled (Andor Technology, Belfast, Northern Ireland) for time-resolved spectra. Samples were aerated and deaerated with nitrogen or oxygen for ca. 15 min before each measurement.

The photoinduced production of singlet oxygen (^1^O_2_) was determined by direct measurement of the luminescence at 1276 nm with an NIR detector integrated in the aforementioned spectrofluorometer (InGaAs detector, Hamamatsu G8605-23). The ^1^O_2_ signal was registered in front configuration (front face), 40° and 50° to the excitation and emission beams, respectively, and leaned 30° to the plane formed by the direction of incidence and registration in cells of 1 cm. The signal was filtered by a low cut-off of 850 nm. ^1^O_2_-generation quantum yield (ϕ^Δ^) was determined using the following equation:

ϕ^Δ^ = ϕ^Δ,r^·(α^r^/α^PS^)·(Se^PS^/Se^r^)

where ϕ^Δ,r^ is the quantum yield of ^1^O_2_ generation for the used reference (2,6-diiodo-3,5-dimethyl-8-methylthioBODIPY, MeSBDP), which was 0.89 in toluene, 0.91 in chloroform, and 0.95 in acetonitrile [49]. Factor α = 1−10^−Abs^ corrects the different amount of photons absorbed by the sample (α^PS^) and reference (α^r^). Factor Se is the intensity of the ^1^O_2_ phosphorescence signal of the sample (Se^PS^) and the reference (Se^r^) at 1276 nm. ^1^O_2_ quantum yields were averaged from at least five concentrations between 10^−6^ M and 10^−5^ M.

### 3.2. Quantum Mechanics Calculations

Ground-state geometries (S_0_ and T_1_) were optimized using a hybrid exchange-correlation functional with the Coulomb-attenuating method (CAM-B3LYP), within Density Functional Theory (DFT), and a triple valence basis set with a polarization function (6-311g*). All the calculations were run without any geometrical constraint, and the geometries were considered to be at minimum energy when the corresponding frequency analysis did not give any negative value. The time-dependent (TD) method at the above detailed calculation level (functional and basis set) was used to simulate the absorption spectra as vertical Franck–Condon transitions. The solvent effect (chloroform) was also simulated during the calculations by the Self-Consistent Reaction Field (SCRF) using the Polarizable Continuum Model (PCM). All calculations were run on the Gaussian 16 software implemented in the “Arina” informatics cluster of the UPV/EHU.

## 4. Conclusions

Synthetically accessible regioselective monoformylation of BODIPY dimers is demonstrated to be a reliable strategy to modulate the balance between fluorescence vs. ^1^O_2_ photo-generation towards the development of valuable fluorescence photosensitizers for photo-theragnosis. However, the chromophoric position to be formylated must be selected depending on the dimer’s typology (localization of the BODIPY-BODIPY linkage and steric hindrance around it). Thus, in sterically hindered 2-8′ BODIPY dimers, monoformylation is not recommended for obtaining fluorescent PSs. In contrast, in unconstrained 2-8′ BODIPY dimers, monoformylation succeeds in increasing the fluorescence response while retaining significant ^1^O_2_ generation. On the other hand, in 3-8′ BODIPY dimers, regioselective monoformylation, especially at the 3´ position, is highly recommended to achieve fluorescent PSs endowed with dual performance. Thus, the conducted spectroscopic study provides the basis for the development of advanced heavy-atom-free BODIPY-dimer-based PSs, where the dual photo-theragnostic activity can be finely modulated by a simple and straightforward regioselective postfunctionalisation on accessible BODIPY dimers to increase ^1^O_2_ production to enhance PDT activity, or to increase the fluorescence signalling to enhance diagnostics capability using fluorescence bioimaging.

Although the prediction of the impact of other types of functionalisation on the photophysical properties of the BODIPY-based dimers is extremely risky, even when selecting substituents with similar stereo-electronic properties. Surely, a possible predictive approach should take into account that the modulation of the dimer photophysics depends on each functionalization and should be optimized in each specific case. The herein reported results support the construction of formylBODIPYs as a successful strategy to modulate the performance of this interesting family of heavy-atom-free PSs via a low-cost and easy chemical modification, regardless of the geometrical arrangement of the starting BODIPY dimer.

## Figures and Tables

**Table 1:** Photophysical properties of non-formylated (**1**–**3**) and formylated (**1a,b**, **2a,b**, and **3a,b**) BODIPY dimers in diluted solutions (2 μM) of chloroform. Full photophysical data in other solvents are listed in Table S1 in Supplementary Materials.

	λ_ab_ (±0.5 nm)	*ε*_max_ * (10^4^ M^−1^ cm^−1^)	λ_fl_ (±0.5 nm)	*ϕ*_fl_ *	τ_fl_ * (ns)	*ϕ*^Δ^*	*τ*^T^ * (μs)
**1**	511.0	16.5	525.0	0.19	0.02 (78%)–5.02 (22%)	0.84	220
**1a**	509.0	6.2	521.5	0.01	1.54 (21%)–4.21 (79%)	0.50	165
**1b**	505.5	13.9	529.5 625.0	0.02	0.02 (94%)–1.65 (6%) 1.23 (38%)–1.91 (62%)	0.75	215
**2**	507.0	6.7	522.5	0.01	1.59 (25%)–4.84 (75%)	0.64	165
**2a**	499.5	5.4	519.0 638.0	0.02	1.85 (21%)–4.87 (79%) 1.18 (92%)–2.62 (8%)	0.62	110
**2b**	507.0	8.7	587.0	0.16	2.59 (32%)–4.36 (68%)	0.50	198
**3**	507.0	7.9	513.5 676.0	0.03	2.59 (15%)–4.36 (85%) 1.43	0.55	110
**3a**	518.0	4.8	559.5	0.19	0.50 (14%)–4.06 (86%)	0.22	-

Absorption (λ_ab_) and fluorescence (λ_fl_) wavelength, molar absorption at the maximum (*ε*_max_), fluorescence quantum yield (*ϕ*_fl_) and fluorescence lifetime (*τ_fl_*), ^1^O_2_ photo-generation quantum yield (*ϕ*^Δ^), triplet-state lifetime (*τ*^T^). * The estimated relative standard deviation (RSD) is less than 5% in *ε*_max,_ *ϕ*_fl_ and *τ_fl_,* and up to 10% in *ϕ*^Δ^ and *τ*^T^.

## Data Availability

The data presented in this study are available in the article or Supplementary Materials.

## Supplementary Materials

The following supporting information can be downloaded at https://www.mdpi.com/article/10.3390/ijms241411837/s1.
