# Supplementary material for: Formylation as a Chemical Tool to Modulate the Performance of Photosensitizers Based on Boron Dipyrromethene Dimers
Source: Int J Mol Sci. 2023 Jul 23;24(14):11837. doi: 10.3390/ijms241411837 (PMC10380594; doi:10.3390/ijms241411837)
Supplement: Supplementary file 1 [file ijms-24-11837-s001.zip › ijms-2507161-supplementary.pdf]

Supplementary Materials

Formylation as a Chemical Tool to Modulate the Performance of Photosensitizers Based on Boron Dipyrromethene Dimers

Carolina Díaz-Norambuena [1,2], Edurne Avellanal-Zaballa [1], Alejandro Prieto-Castañeda [1,2], Jorge Bañuelos [1,*], Santiago de la Moya [2], Antonia R. Agarrabeitia [2,3] and María J. Ortiz [2,*]

1 Departamento de Química Física, Facultad de Ciencia y Tecnología, Universidad del País Vasco-EHU,
Apartado 644, 48080 Bilbao, Spain; carolina.diaz@ehu.eus (C.D.-N.); edurne.avellanal@ehu.eus (E.A.-Z.); alejandro.prietoc@ehu.eus (A.P.-C.)

2 Departamento Química Orgánica, Facultad de Ciencias Químicas, Universidad Complutense de Madrid, Ciudad Universitaria s/n, 28040 Madrid, Spain; santmoya@ucm.es (S.d.l.M.); agarrabe@quim.ucm.es (A.R.A.)

3 Sección Departamental de Química Orgánica, Facultad de Óptica y Optometría, Universidad Complutense de Madrid, Arcos de Jalón 118, 28037 Madrid, Spain

* Correspondence: jorge.banuelos@ehu.es (J.B.); mjortiz@quim.ucm.es (M.J.O.)

Table S1. Photophysical data S2

Figure S1. Absorption and fluorescence spectra of dimers 2-8′ S3

Figure S2. S_0 optimized geometries and frontier MOs of dimers 2-8′ S4

Figure S3. Absorption and fluorescence spectra of dimers 3-8′ S5

Figure S4. S_0 optimized geometries and frontier MOs of dimers 3-8′ S6

Figure S5. Absorption and fluorescence spectra of unconstrained dimers 2-8′ S7

Figure S6. S_0 optimized geometries and frontier MOs of unconstrained dimers 2-8′ ... S8

Figure S7. Ns-transient absorption spectra S9

Figure S8. T_1 optimized geometries and spin densities S10

Table S1. Photophysical properties of the BODIPY-based 2-8′ and 3-8′ dimers and their formylated derivatives in diluted solutions (2 μM) of different solvents.

		λ_{ab} (nm)	$\varepsilon_{máx}$ (10^{-4} $M^{-1}cm^{-1}$)	λ_{fl} (nm)	ϕ	τ (ns)	ϕ^{Δ}
**1**	Toluene	511.0	17.3	525.0	0.46	1.56(5%) - 5.87(95%)	0.41
	$CHCl_3$	511.0	16.5	525.0	0.19	0.02(78%) - 5.02(22%)	0.84
	ACN	505.0	15.4	512.5	0.03	0.63(10%) - 4.67(90%)	0.65
**1a**	Toluene	511.5	6.7	545.5	0.030	1.88(40%) - 4.30(60%)	0.93
				605.0		1.88(93%) - 4.79(7%)	
	$CHCl_3$	509.0	6.2	521.5	0.011	1.54(21%) - 4.21(79%)	0.50
	ACN	504.5	5.5	513.0	0.005	-	0
**1b**	Toluene	509.0	14.7	534.0	0.175	0.89(9%) - 3.32(91%)	0.71
	$CHCl_3$	505.5	13.9	529.5	0.022	0.02(94%) - 1.65(6%)	0.75
				625.0		1.23(38%) - 1.91(62%)	
	ACN	501.5	12.9	517.0	0.002	-	0
**2**	Toluene	509.0	7.3	529.0	0.021	1.27(17%) - 4.49(83%)	0.84
				596.0		1.51(25%) - 4.01(75%)	
	$CHCl_3$	507.0	6.7	522.5	0.011	1.59(25%) - 4.84(75%)	0.64
	ACN	506.0	6.5	518.0	0.004	-	0
**2a**	Toluene	504.5	7.2	589.5	0.24	4.19	0.61
	$CHCl_3$	499.5	5.4	519.0	0.020	1.85(21%) - 4.87(79%)	0.62
				638.0		1.18(92%) - 2.62(8%)	
	ACN	496.5	5.4	527.5	0.005	-	0
**2b**	Toluene	510.0	8.9	586.5	0.268	2.06(18%) - 5.49(82%)	0.40
	$CHCl_3$	507.0	8.7	587.0	0.161	2.59(32%) - 4.43(68%)	0.50
	ACN	502.5	8.0	517.5	0.003	-	0.23
**3**	Toluene	509.5	8.8	518.5	0.144	2.89 (44%) - 5.49 (56%)	0.80
				580.0		1.02 (10%) - 2.50 (90%)	
	$CHCl_3$	507.0	7.9	513.5	0.028	2.59 (15%) - 4.36 (85%)	0.55
				676.0		1.43	
	ACN	502.5	7.9	508.0	0.004	-	0
**3a**	Toluene	521.5	5.1	558.5	0.348	1.91 (11%) - 5.97 (89%)	0.31
	$CHCl_3$	518.0	4.8	559.5	0.189	0.50 (14%) - 4.06 (86%)	0.22
	ACN	508.0	4.6	508.5	0.004	-	0.17

Absorption (λ_{ab}) and fluorescence (λ_{fl}) wavelength, molar absorption at the maximum (ε_{max}), fluorescence quantum yield (ϕ) and lifetime (τ), and singlet oxygen generation quantum yield (ϕ^{Δ}).

$CHCl_3$: chloroform; ACN: acetonitrile

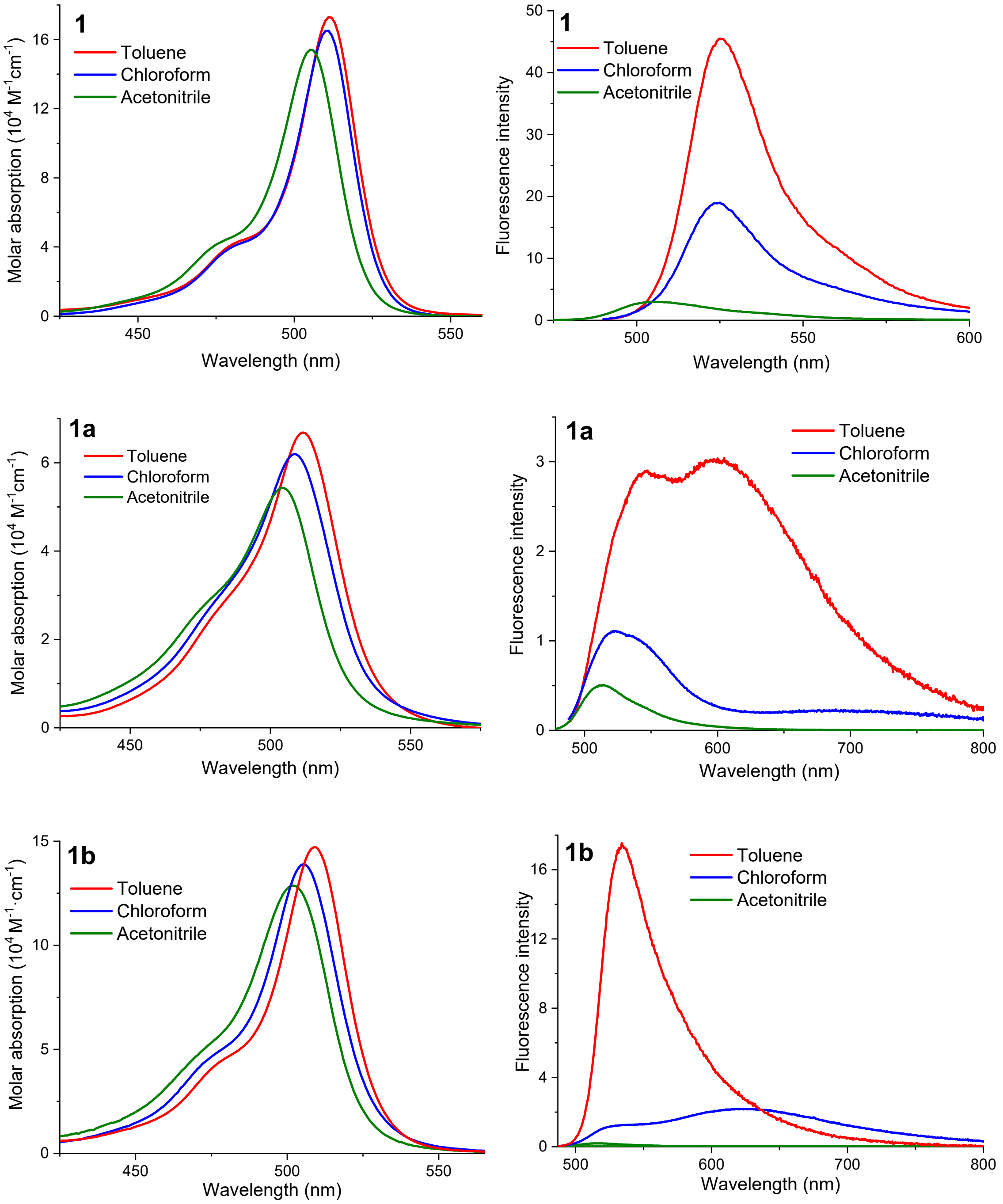

Figure S1. Absorption and fluorescence (scaled by fluorescence quantum yield) spectra of the 2-8′ dimers in diluted solutions of different solvents.

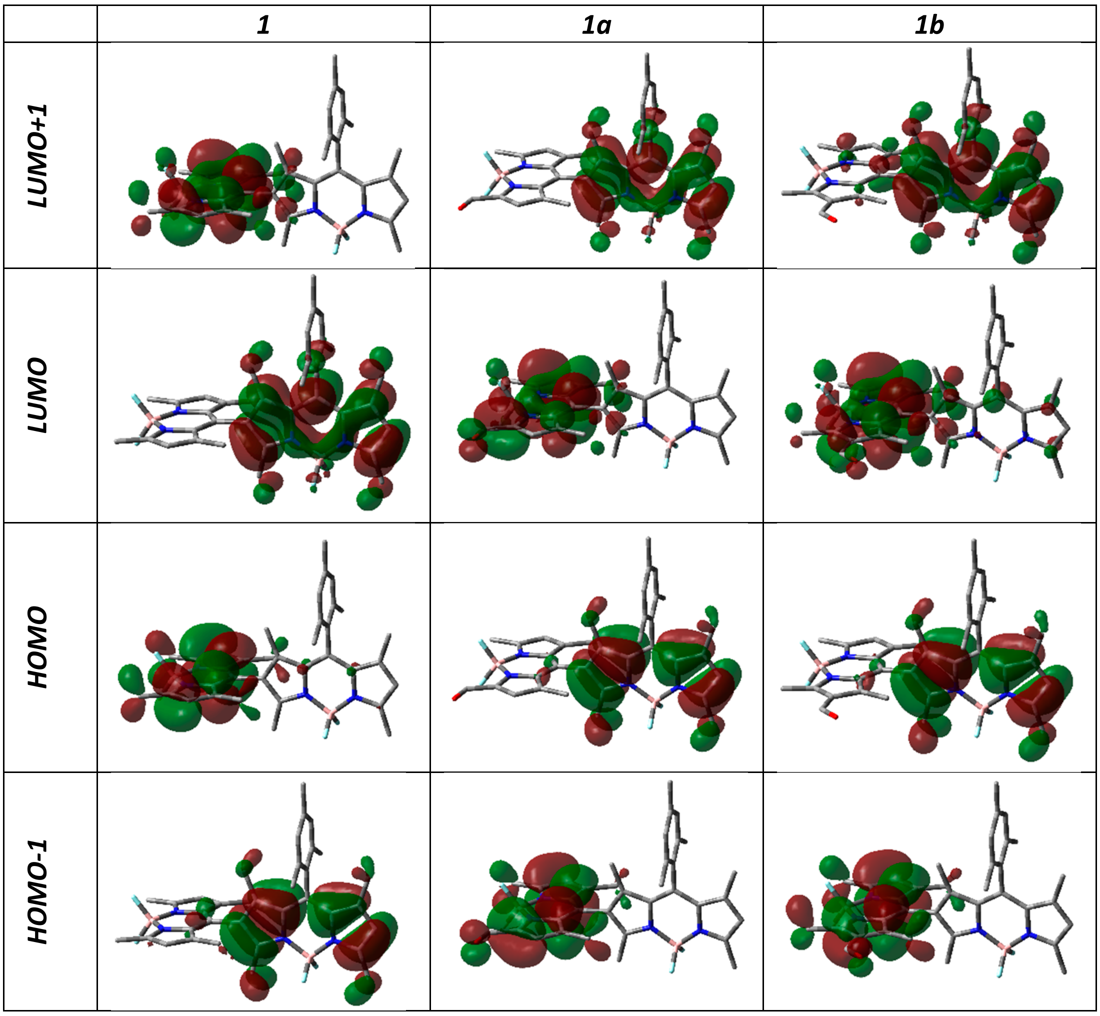

Figure S2. Contour maps of the main molecular orbital involved in the absorption transition of the 2-8′ dimers attained from their ground state optimized geometries (CAM-B3LYP/6-311g*) in chloroform (PCM).

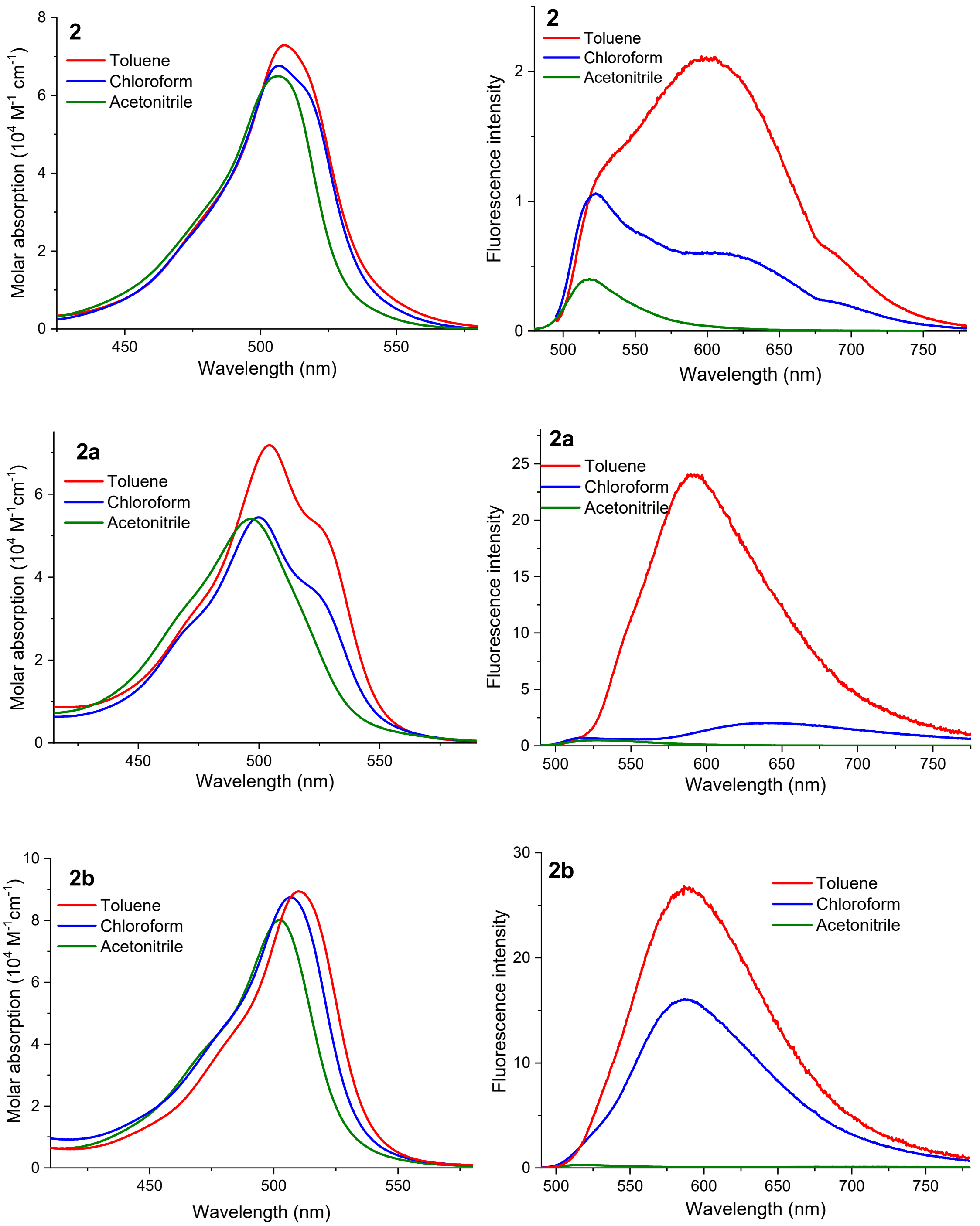

Figure S3. Absorption and fluorescence (scaled by fluorescence quantum yield) spectra of the 3-8′ dimers in diluted solutions of different solvents.

	2	2a	2b
LUMO+1	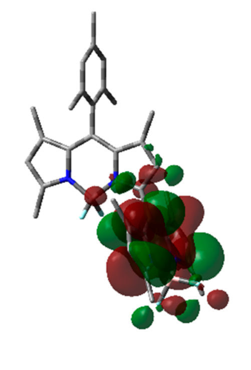	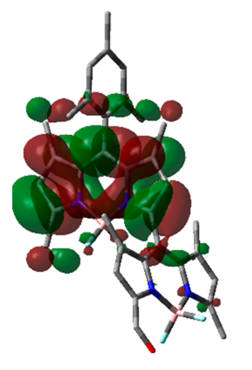	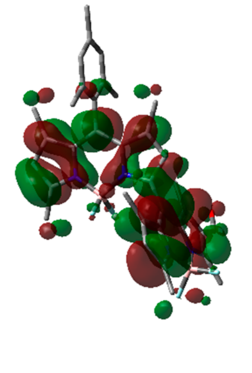
LUMO	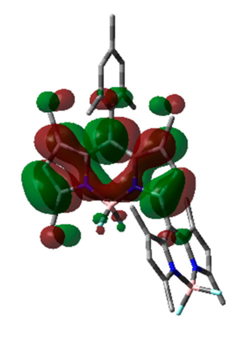	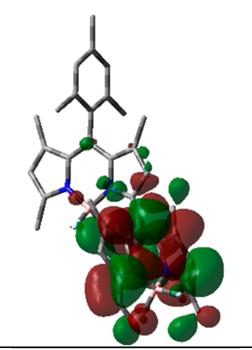	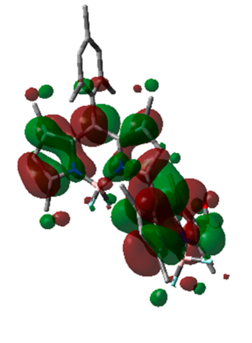
HOMO	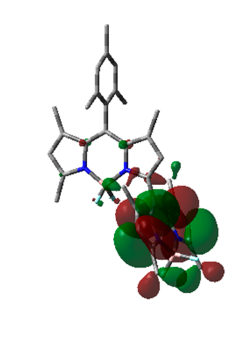	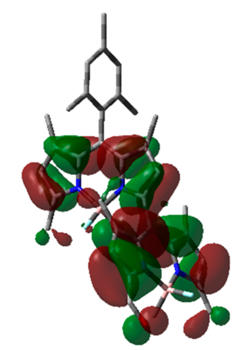	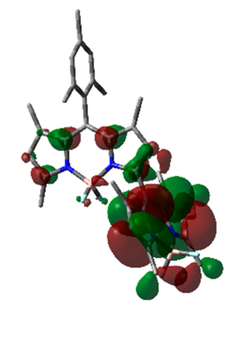
HOMO+1	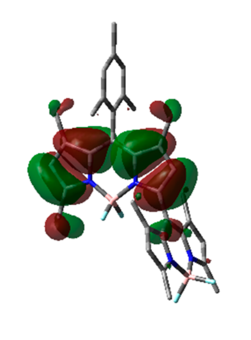	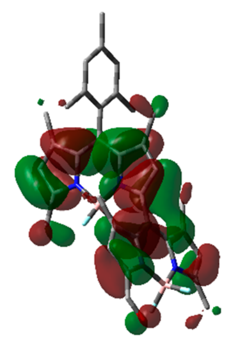	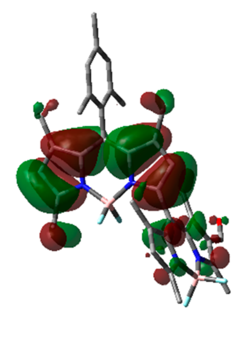

Figure S4. Contour maps of the main molecular orbital involved in the absorption transition of the 3-8′ dimers attained from their ground state optimized geometries (CAM-B3LYP/6-311g*) in chloroform (PCM).

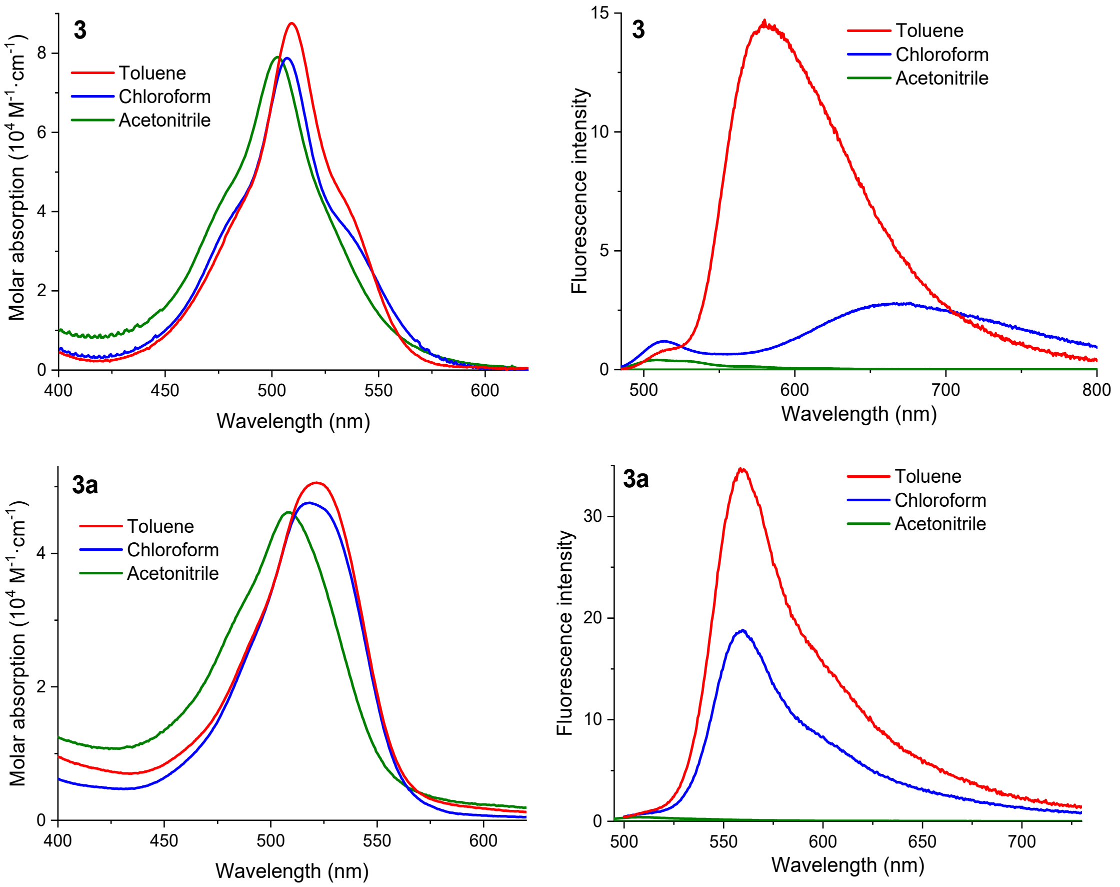

Figure S5. Absorption and fluorescence (scaled by fluorescence quantum yield) spectra of dimer **3** and its 3-formylated derivative **3a** in diluted solutions of different solvents.

	3	3a
LUMO+1	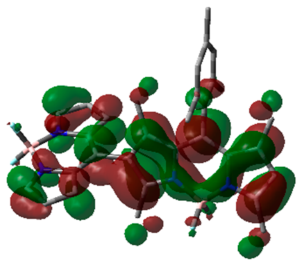	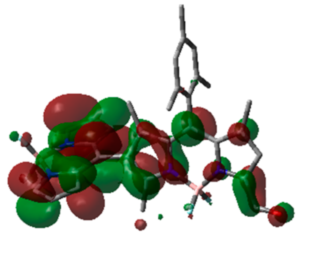
LUMO	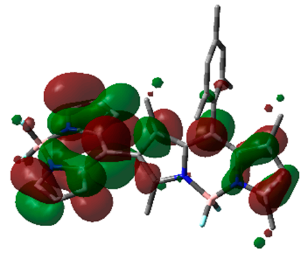	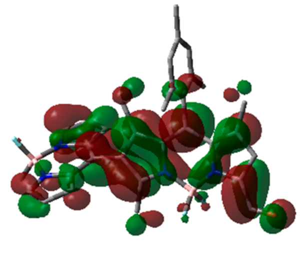
HOMO	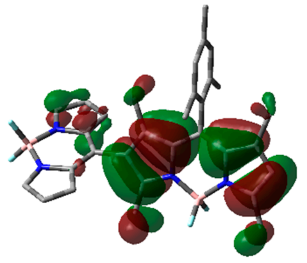	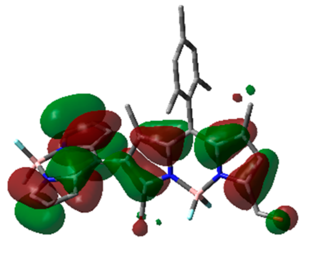
HOMO-1	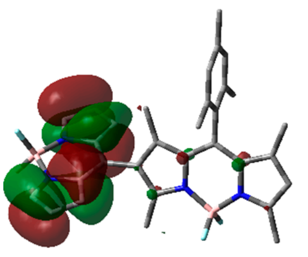	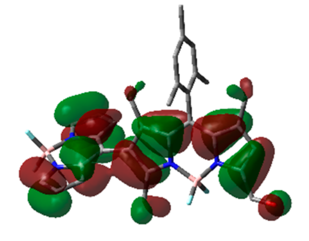

Figure S6. Contour maps of the main molecular orbital involved in the absorption transition of the less constrained 2-8′ dimers attained from their ground state optimized geometries (CAM-B3LYP/6-311g*) in chloroform (PCM).

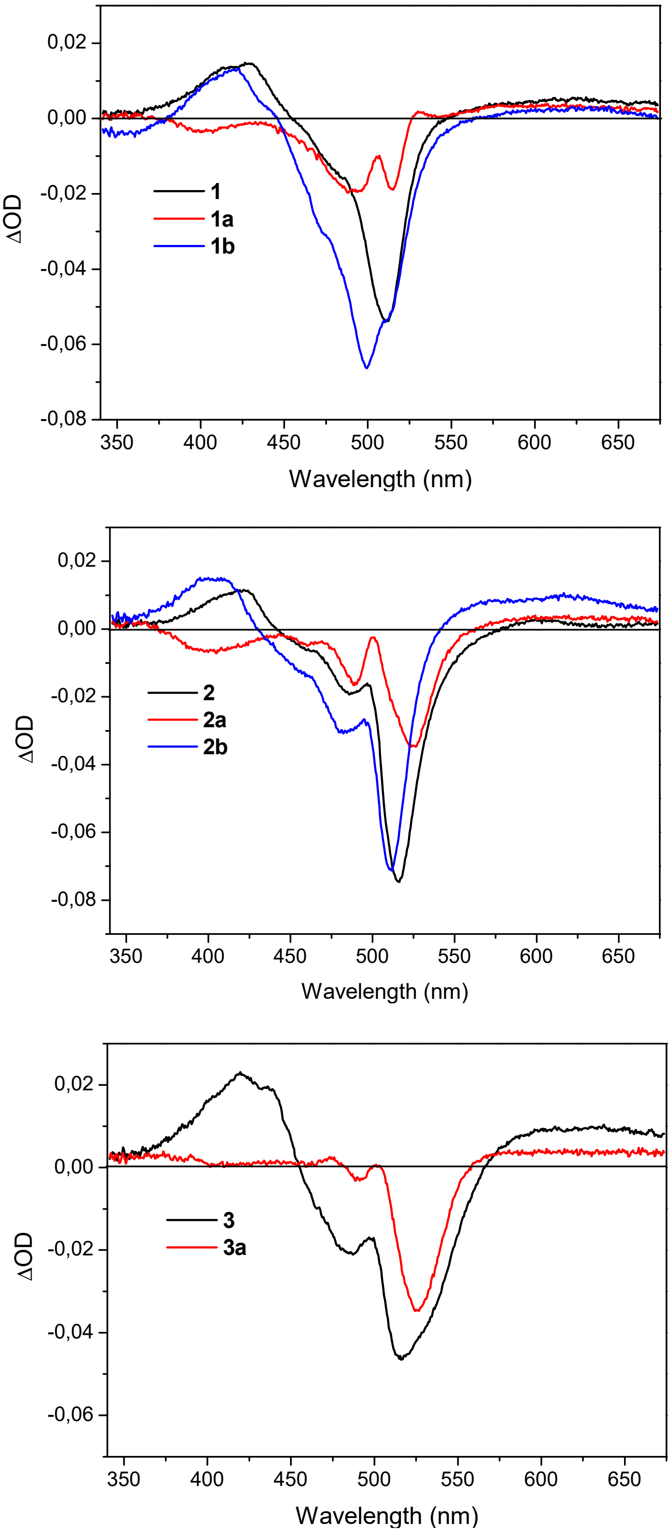

Figure S7. Transient absorption spectra of the dimers (10^{-5} M) in chloroform solutions after purging them with nitrogen during 15 minutes.

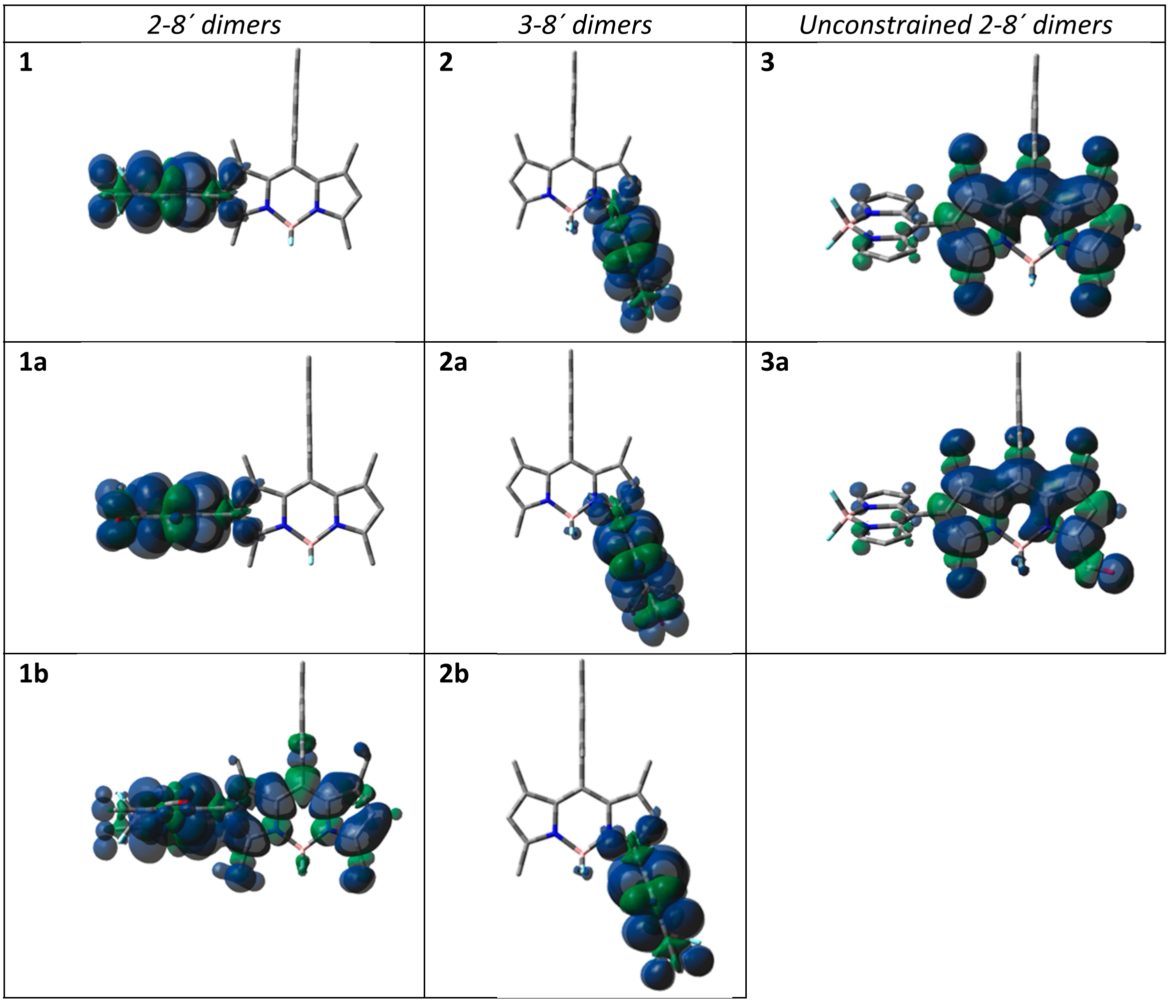

Figure S8. Isosurfaces of spin-density of the optimized T_1 state geometry (CAM-B3LYP/6-311g*) for all the tested dimers in chloroform (PCM).
